# Identification, functional characterization, assembly and structure of ToxIN type III toxin–antitoxin complex from *E. coli*

**DOI:** 10.1093/nar/gkab1264

**Published:** 2022-01-08

**Authors:** Parthasarathy Manikandan, Sankaran Sandhya, Kavyashree Nadig, Souradip Paul, Narayanaswamy Srinivasan, Ulli Rothweiler, Mahavir Singh

**Affiliations:** Molecular Biophysics Unit, Indian Institute of Science, Bengaluru 560012, India; Molecular Biophysics Unit, Indian Institute of Science, Bengaluru 560012, India; Molecular Biophysics Unit, Indian Institute of Science, Bengaluru 560012, India; Molecular Biophysics Unit, Indian Institute of Science, Bengaluru 560012, India; Molecular Biophysics Unit, Indian Institute of Science, Bengaluru 560012, India; The Norwegian Structural Biology Centre, Department of Chemistry, The Arctic University of Norway, N-9037 Tromsø, Norway; Molecular Biophysics Unit, Indian Institute of Science, Bengaluru 560012, India

## Abstract

Toxin–antitoxin (TA) systems are proposed to play crucial roles in bacterial growth under stress conditions such as phage infection. The type III TA systems consist of a protein toxin whose activity is inhibited by a noncoding RNA antitoxin. The toxin is an endoribonuclease, while the antitoxin consists of multiple repeats of RNA. The toxin assembles with the individual antitoxin repeats into a cyclic complex in which the antitoxin forms a pseudoknot structure. While structure and functions of some type III TA systems are characterized, the complex assembly process is not well understood. Using bioinformatics analysis, we have identified type III TA systems belonging to the ToxIN family across different *Escherichia coli* strains and found them to be clustered into at least five distinct clusters. Furthermore, we report a 2.097 Å resolution crystal structure of the first *E. coli* ToxIN complex that revealed the overall assembly of the protein-RNA complex. Isothermal titration calorimetry experiments showed that toxin forms a high-affinity complex with antitoxin RNA resulting from two independent (5′ and 3′ sides of RNA) RNA binding sites on the protein. These results further our understanding of the assembly of type III TA complexes in bacteria.

## Graphical Abstract

**Graphical Abstract:**
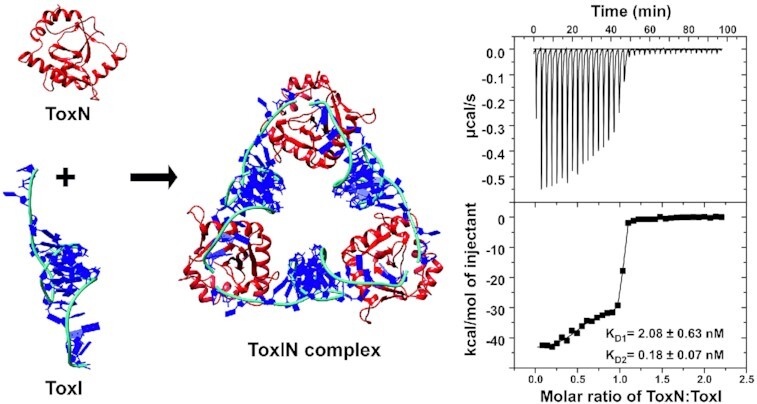
Structure and assembly of the first *E. coli* type III toxin–antitoxin complex.

## INTRODUCTION

Toxin-antitoxin (TA) systems are being understood as a key bacterial defense mechanism against invading viruses, antibiotics, and other environmental stress. TA systems consist of a pair of genes, under a common promoter, that code for a toxin and its cognate antitoxin (1,2) The toxin is usually a protein that arrests cell growth during stress, whereas the antitoxin can be a protein or a noncoding RNA that inhibits the toxin's activity. The TA systems are classified into mainly six different types based on the mechanism of inhibition of toxin by antitoxin (1,2). Type III TA systems were initially described as phage abortive infection systems and later recognized as toxin–antitoxin systems (3–5). In type III TA systems, the toxin is an endoribonuclease (RNase) that cleaves cellular RNAs when free, whereas antitoxin is a noncoding RNA. The toxin also processes its precursor antitoxin RNA into smaller repeats and subsequently assembles with the individual repeats to form an inactive TA complex (6).

The type III TA complexes are unique self-closing RNA–protein assembly, in which the toxin and antitoxin are arranged alternately in a 1:1 ratio (6–8). The mechanism by which the complex assembles into a cyclic multimer from individual units is not completely clear. Moreover, during stress such as phage infection, the TA systems are activated with the release of active toxins. The free toxin in the cell cleaves its target cellular RNAs and thereby causes bacterial growth arrest (6). Therefore, specific activation of TA systems in pathogenic bacteria has been proposed as a novel antibacterial strategy (9). However, a molecular mechanism of activation of type III TA systems is not well understood. The structure of a few type III TA complexes has been determined and their cellular functions have been deciphered (6–8). However, their assembly process is not well understood in quantitative terms, such as the affinity of toxin for antitoxin RNA in TA complex.

Type III TA systems are classified into three different families: ToxIN, CptIN, and TenpIN (I – antitoxin RNA, N – toxin protein), based on protein sequence identity (10). Although type III TA systems have been identified and classified in several bacteria, these systems were not well characterized in *Escherichia coli*. Very recently, while this work was in preparation, Guegler and Laub reported functional characterization of the first *E. coli* to type III TA system from *E. coli* GCA_001012275 (11). This work provided very interesting and new insights into the functioning of type III TA systems. The results showed that upon activation of the type III TA system due to phage infection in *E. coli*, the TA system inhibits the production of new virions by directly cleaving the viral RNA transcripts. Further studies will likely show the cellular or phage factors that can directly interact with the type III TA systems to control its activity.

In this study, we report the identification of type III systems in different *E. coli* strains belonging to the ToxIN family. Based on homology searches, we found that the ToxIN systems in various *E. coli* strains may be grouped into five distinct clusters. Toxin ToxN proteins in a particular cluster shared a high level of sequence identity, and the sequence and length of antitoxin ToxI RNA repeats seem to be unique for each cluster. This analysis has also revealed a set of highly conserved residues in ToxN, which are likely to be important for the function and structure of the toxin. We have further cloned one ToxIN system from *E. coli* strain 680 and functionally characterized it. Expression of ToxN alone led to inhibition of bacterial growth that was alleviated when ToxN was co-expressed with cognate ToxI RNA. Furthermore, we have co-expressed and co-purified the toxin and antitoxin components. The *E. coli* ToxIN complex was successfully crystallized, and the structure was solved at 2.097 Å resolution. The structure revealed the molecular architecture of the first *E. coli* ToxIN complex. Importantly, we have studied the binding of ToxN with antitoxin ToxI repeats using the isothermal titration calorimetry (ITC) method. ITC experiments revealed that protein toxin forms a high-affinity complex with antitoxin RNA resulting from two independent (5′ and 3′ sides of RNA repeats) RNA binding sites on the protein. These results provide key insights into the mechanism of assembly of type III TA complexes. NMR experiments on complete and truncated ToxI repeats suggested that free ToxI is folded and forms a pseudoknot structure without ToxN.

## MATERIALS AND METHODS

### Identification of type III toxins in *Escherichia coli*

ToxIN and AbiQ type III toxin sequences were employed as queries in homology searches against the non-redundant databank (NRDB) (12) to probe for homologues in *E. coli*. For this, representative ToxN sequences of Q3YN09 (PDB ID 4ATO, 194 residues, *Bacillus thuringiensis*), B8X8Z0 (PDB ID 2XDD, 171 residues, *P. atrosepticum*), Q9ZJ19 (PDB ID 4GLK, 183 residues, *Lactococcus lactis*) of the ToxIN and AbiQ type III TA were first obtained from the UniProt database (13). BlastP searches were carried out in the NRDB to identify hits in *E. coli* at an *E*-value <0.0001 and query coverage >60% of query length, using the BLOSUM-45 substitution matrix (14). Although multiple queries were employed to probe for homologues, there was considerable overlap in hits from the searches. Therefore, toxin hits from the independent searches were pooled together and their sequences were further analyzed.

Since type III antitoxins are RNA molecules, cognate antitoxin sequences for each of the predicted toxins were obtained by probing the genomic region upstream of the putative toxin sequences. The coding sequence and 1 kb up-and downstream were extracted from the NCBI database for each protein. Specifically, we probed for the −10 and −35 promoter sequence signatures upstream of the predicted coding sequence of the toxin. The sequence between the −10 residue and the start of the toxin gene was probed for the presence of potential repeats. Repeats were recognized manually and verified using Tandem Repeat Finder (15). Default settings were used for the searches (match, mismatch, indels = 2, 7, 7; min score = 50). After the repeats were identified, their size, number, and potential to form pseudoknots were determined. Potential pseudoknots were predicted using vsfold5 (16) and IPKnot (17). Terminator sequences were recognized using the Mfold tool (18).

### Multiple sequence alignments of putative toxin sequences and antitoxin repeats from *E. coli*

We aligned the toxin sequences of homologues from various strains of *E. coli* to compare them using MAFFT-DASH (19). Structure-guided alignments were also performed using Promals3D to assess conservation in ToxN sequences (20). Alignments were visualized using Espript (21). Residues known to lie at the toxin–antitoxin interface were obtained from the known structural templates and mapped to the alignment to study the conservation of such residues in the homologues. To generate a representative alignment, ten representative ToxN sequences from the five clusters (two from each cluster) were aligned using COBALT (22) and the alignments were visualized using Espript (21). Alignment of ToxI repeats (one repeat from each cluster) was performed manually based on the single functional repeat of ToxIN_Ec_ as observed in the crystal structure. The 5′ and 3′ termini of ToxI in each cluster were derived based on the *E. coli* and *P. atrosepticum* ToxI repeat alignments, as the information about the consensus cleavage site of their corresponding ToxNs is not available.

### Bacterial strains, plasmids, cloning, and site-directed mutagenesis

The Type III TA operon from *E. coli* (strain 680, assembly GCA_001893605.1), including natural promoter, was synthesized and cloned into a pUC57 vector by GenScript (USA). The antitoxin sequence along with natural promoter and terminator regions was amplified using primers EctaiiiRNA_Fwd and EctaiiiRNA_Rev and cloned into pRSFDuet™-1 vector modified using primers pRSF_Fwd and pRSF_Rev between restriction sites NcoI and XhoI (Supplementary Table S1). The toxin gene was cloned into pCold™ II vector using primers Ectoxin_Fwd and Ectoxin_Rev between restriction sites NdeI and XbaI to give an N-terminal hexahistidine (6X-His) affinity purification tag (Supplementary Table S1). The positive toxin clones were obtained by co-transforming the ligation product with an antitoxin-containing plasmid. For the toxicity assays, the untagged toxin gene was cloned into pBAD/His A vector between restriction sites NcoI and XhoI. The positive clones were confirmed by sequencing. *E. coli* DH5α cells were used for cloning, and *E. coli* BL21(DE3) cells were used to express the TA complex. Cells were grown in LB media supplemented with 50 mg/ml kanamycin, 100 mg/ml ampicillin, 1 mM IPTG, 0.2% D-glucose, and 0.2% l-arabinose. ToxN_Ec_ single-site mutants were generated by site-directed mutagenesis on the pBAD/His A vector containing the WT ToxN gene using appropriate primers (Supplementary Table S1).

### Toxin-antitoxin functional growth assays

For functional assays, untagged toxin protein cloned in the pBAD/His A vector and antitoxin along with natural promoter and terminator cloned in modified pRSFDuet™-1 were used. pBAD/His A empty vector was used as control. Primary cultures of *E. coli* DH5α cells were grown with empty vector, toxin or both toxin and antitoxin containing plasmids overnight in LB media containing D-glucose and appropriate antibiotics (ampicillin for empty vector and ampicillin + kanamycin for toxin and antitoxin together) at 37°C, 180 rpm to repress the P_ara_BAD promoter. A secondary culture was grown under the same conditions until the optical density at 600 nm wavelength (OD_600_) reached ∼0.2. Subsequently, the cells were pelleted and resuspended in LB media containing l-arabinose and appropriate antibiotics and grown at 37°C, 180 rpm and OD_600_ was monitored every 30 min and plotted on a log scale. For colony counting experiments, the cells were pelleted down after 2.5 h of arabinose induction, washed and resuspended to a final OD_600_ ∼1. The cultures were serially diluted and plated on LB agar plates containing D-glucose and appropriate antibiotics. The plates were incubated at 37°C overnight for cell growth and the colony counts were obtained. For serial dilution assays, the saturated primary culture was resuspended in LB media containing l-arabinose and appropriate antibiotics and spotted as serial dilutions in LB agar plates, containing l-arabinose and appropriate antibiotics and incubated at 37°C overnight. Serial dilution experiments of toxin mutants were also carried out similarly.

### Expression and purification of the complex, toxin, and antitoxin

The plasmids containing toxin and antitoxin were co-transformed into *E. coli* BL21(DE3) cells and grown overnight at 37°C, 180 rpm, followed by the secondary culture at 37°C, 180 rpm till OD_600_ ∼0.5. The culture was incubated at 15°C without shaking for 30 min and the toxin was induced by adding IPTG to a final concentration of 1 mM and incubated at 15°C, 180 rpm for 24 h. The cells were harvested by centrifugation at 6000 rpm for 15 min. The cells were resuspended in lysis buffer (50 mM Tris, 300 mM NaCl, 10 mM imidazole, 10% glycerol, 2 mM 2-mercaptoethanol pH 7.5 at 25°C) and lysed by sonication. The lysate was centrifuged at 13 000 rpm for 30 min, and the supernatant was loaded on a Ni^2+^-NTA column. The complex was eluted using elution buffer (lysis buffer + 200 mM imidazole). Fractions containing the complex were dialyzed against ion-exchange buffer (50 mM NaCl, 50 mM Tris–HCl, 1 mM DTT pH 7.5) and purified using anion exchange chromatography by increasing gradient of NaCl from 50 to 1000 mM, over a volume of 100 ml, which yielded separate fractions of toxin (at ∼300 mM NaCl), antitoxin (at ∼600 mM NaCl) and complex (at ∼500 mM NaCl). They were further purified by size exclusion chromatography (SEC) using an S200 column (GE).

### SEC-MALS

The molecular mass of the ToxIN complex in solution was determined using SEC-MALS. The experiment was performed on a Shimadzu chromatography system consisting of a miniDAWN TREOS MALS detector and a WATERS 2414 refractive index (RI) detector. The system was calibrated using bovine serum albumin (BSA). The experiment was performed by passing 100 μL of SEC purified, centrifuged ToxIN complex through GE S200 (10/300) column. The data was analysed using ASTRA VI software (Wyatt Technology) and the molecular mass was obtained.

### Complex crystallization and structure determination

The ToxIN complex containing fractions after SEC were concentrated to ∼10 mg/ml and used in crystallization trials. Initial screens were carried out using Natrix-HT (Hampton) and Nucleix Suite (Qiagen) in 72-well oil immersion plates. The ToxIN complex crystals were further optimized using the hanging drop vapor diffusion method. The crystals for the ToxIN complex appeared within a week at 20°C in 0.1 M disodium succinate pH 5.5, 1.5 mM spermidine, 0.02 M MgCl_2_, and 2.4 M ammonium sulfate. The X-ray diffraction data sets were collected at synchrotron Helmholtz-Zentrum Berlin, Germany (BESSY II). The crystals diffracted up to a maximum resolution of 2.097 Å. The diffraction data sets were processed by iMosflm and XDSAPP software (23–25). The structure was solved by the Molecular Replacement method using the ToxIN complex structure from *P. atrosepticum* (PDB 2XDB) as the search model. Coot and Phenix were used for iterative model building and refinement (26,27). The *R*_work_ and *R*_free_ of the final model of the ToxIN_Ec_ heterohexamer complex are 0.212 and 0.232, respectively (Table 1). The model quality was examined using MolProbity utility of the PHENIX validation suite (27,28).

**Table 1.** Crystallographic data collection and structure refinement statistics

PDB ID	7D8O
**Integration**	
Space group	*P* 1 2_1_ 1
Cell constants	*a* = 86.630 Å, *b* = 86.643 Å, *c* = 123.568 Å α = 90.00°, β = 91.66°, γ = 90.00°
Wavelength (Å)	0.9184
Observed reflections	364 098
Unique reflections	104 879
CC(1/2) %	99.3
% Data completeness (in resolution range)	97.51 (43.297–2.097)
<*I*/σ(*I*)>	1.17 (at 2.097 Å)
Resolution range (Å)	43.297–2.097
**Refinement**	
No. of reflections	104 548
*R*_work_, *R*_free_	0.213, 0.232
*R*_free_ test set	2096 reflections (2.00%)
Average *B*, all atoms (Å^2^)	37
R.m.s.d. bond length (Å)/angles (°)	0.004/0.822
Total number of atoms	Total: 13 509 Solvent: 498 Non-solvent: 13 011
Ramachandran outliers allowed/generous/disallowed (%)	99.07/0.83/0
MolProbity clash score (percentile rank)	2.6 (99^th^)

### RNA *in vitro* transcription and purification

The ToxI RNA variants for ITC, NMR and endoribonuclease experiments were *in vitro* transcribed using corresponding DNA templates (Supplementary Table S1) and T7 RNA polymerase. The transcribed RNAs were ethanol precipitated, and the resulting RNA pellets were dissolved in Milli-Q H_2_O and purified using urea-TBE denaturing PAGE. The gel was visualized under UV, and the desired bands were excised from the gel. The RNAs were extracted from the excised gel pieces by electroelution using 1X TBE buffer. The RNAs were further purified by anion exchange chromatography on a 5 ml Hi-Trap Q HP column. The resulting RNAs were exchanged extensively into water using a Centricon device (Millipore). The RNAs were heated to 95°C for 3 min and snap cooled in ice for 25 min and used in further experiments. All RNAs prepared by *in vitro* transcription were synthesized such that they start with GG dinucleotides at their 5′ end to increase the efficiency of transcription.

### ITC experiments

Isothermal titration calorimetry experiments were performed using a VP-ITC machine (MicroCal, USA) at 15°C. The toxin and antitoxin were purified by SEC in ITC buffer (50 mM KH_2_PO_4_ pH 7, 100 mM KCl) before being used in ITC experiments. The RNA and protein were quantified by measuring UV *A*_260 nm_ and *A*_280 nm_ values, respectively. The sample cell was filled with 2–5 μM of antitoxin RNA and titrated with 35–50 μM of toxin protein in the syringe. The ITC experiments of ToxN with ToxI-DNA was also performed using the aforementioned protocol. The integrated heat data was adjusted for the heat of dilution and was fit to a two-site binding model for ToxI and one site binding model for Δ3′-ToxI and Δ5′-ToxI using ORIGIN-5 software provided by the manufacturer.

### NMR spectroscopy

ToxI repeat and 27mer RNAs (0.2–0.6 mM in concentration) were exchanged into NMR buffer (10 mM KH_2_PO_4_ pH 6.3 + 50 mM KCl + 10% D_2_O). 1D 1,1-echo NMR spectra of the imino region of ToxI and 27mer RNAs were recorded at 298 K either on Bruker 700 MHz or 800 MHz spectrometers. The NMR spectra were processed and analyzed using Bruker TopSpin.

### *In vitro* endoribonuclease assay

5 μM of the dimer-ToxI RNA substrate was incubated with different concentrations of ToxN protein (50 nM–15 μM) in the ITC buffer at 37°C for 30 min. The reaction was quenched by adding 2× formamide RNA loading dye and heated to 95°C for 5 min. The samples were analyzed on urea-PAGE (7 M urea, 15% acrylamide) and stained for RNA by using 0.2% toluidine blue solution.

## RESULTS

### ToxIN type III toxin–antitoxin systems are found in several strains of *E. coli*

Type III TA systems have been observed in several bacteria and have been further classified into three families, namely ToxIN, CptIN and TenpIN (10). In this study, we have employed ToxIN sub-type queries of known structures to probe for homologues in the *E. coli* genome. These queries identified 62 homologues in multiple *E. coli* strains (Supplementary Table S2 and Figure S1). Fourteen hits bear a Refseq identifier and point to identical sequences from various strains of *E. coli*. All the hits were identified either on the chromosome or the plasmid in various *E. coli* strains.

To determine if the putative ToxN homologues are functional Type III TA loci, several criteria were employed based on the features of known type III TA operon systems (4–6,29). We probed the 5′ upstream genomic region for the following elements: (i) the presence of a promoter region with characteristic −10 and −35 promoter signatures; (ii) a tandem array of nucleotide repeats downstream of the −10 sequence that would code for the antitoxin repeats (recognized manually and verified computationally using Tandem Repeat Finder) (15); (iii) the presence of a short hairpin forming transcriptional terminator sequence between the antitoxin ToxI repeats and the start of the ToxN protein and (iv) start codon followed by toxin open reading frame. This assessment was performed for every homologue identified in the various strains, and the results are summarized in Supplementary Table S2.

**Figure 1.**
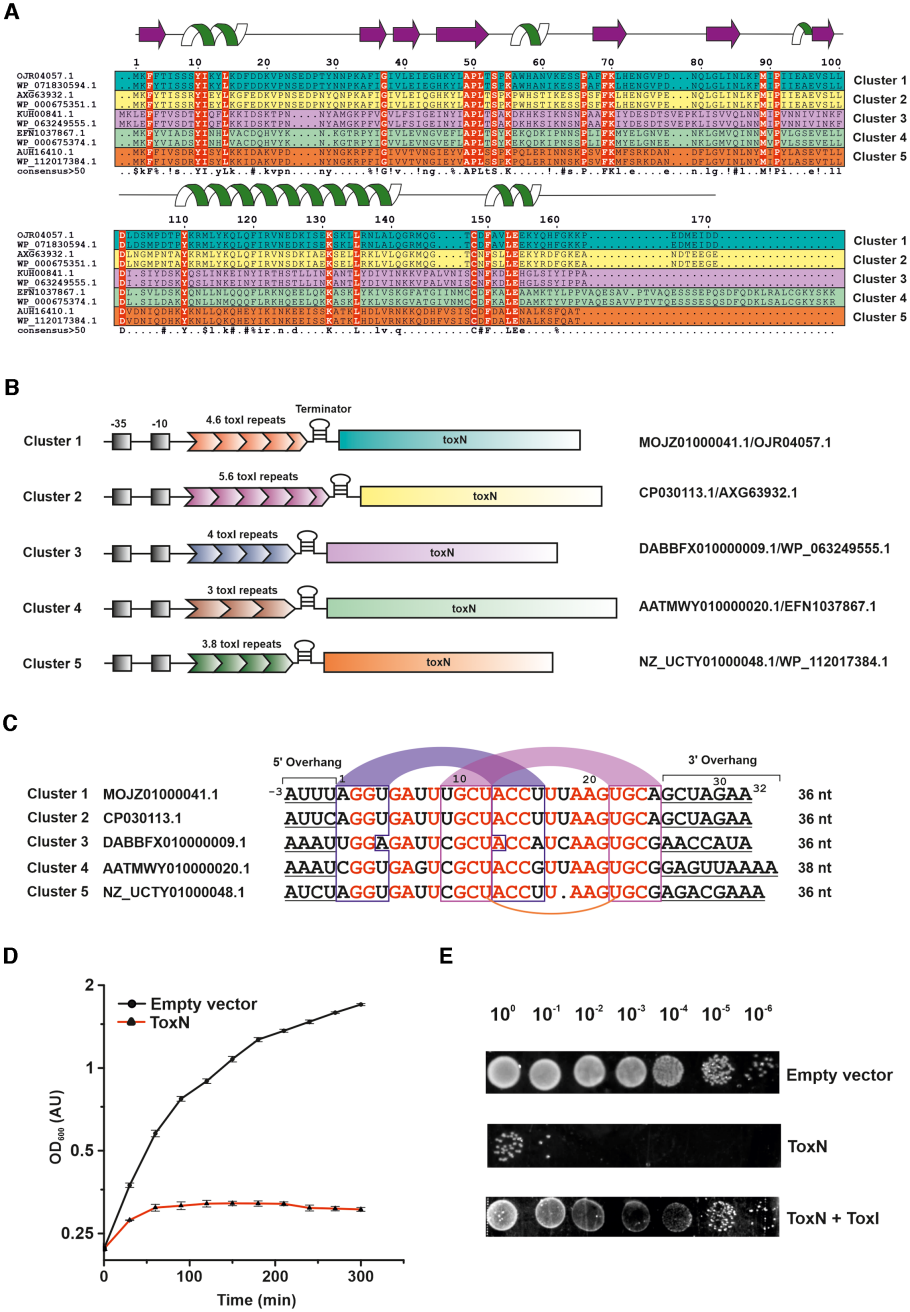
Identification, classification, and functional characterization of type III TA systems from *E. coli*. (**A**) Multiple sequence alignment of representative ToxN sequences belonging to five different clusters (two sequences from each cluster, denoted in five different colours: teal, yellow, magenta, green, and orange) in *E. coli*. The sequence identifiers of each ToxN protein are indicated on the left. The residues conserved in all sequences are denoted in red colour. The secondary structure of ToxN as observed in the ToxIN_Ec_ complex structure from cluster 1 solved in this study, has been depicted at the top of the figure. (**B**) Schematic representation of the ToxIN loci of representatives from the five clusters. The promoter regions (−35 and −10 sequences) of the ToxIN operons are denoted in black shaded boxes. The nucleotide and protein sequence identifiers of each representative locus are indicated on the right. (**C**) Sequence alignment of ToxI repeats representing the five clusters (one from each cluster). The sequence identifiers corresponding to each nucleotide sequence are indicated on the left. The nucleotide bases conserved throughout the alignment are in red. The putative base-pairing regions of stem 1 and stem 2 of the ToxI RNA pseudoknot are in blue and magenta arcs respectively. The conserved uridine bases that form noncanonical U:U:G base triplet in the cluster 1 ToxI pseudoknot have been connected using an orange arc. (**D**) Bacterial growth assay shows that ToxN_Ec_ causes cellular growth arrest upon induction in *E. coli* DH5α cells. (E) 10-fold serial dilutions of *E. coli* DH5α cells expressing ToxN_Ec_, ToxN_Ec_ + ToxI_Ec_ and empty vector control.

We aligned the homologous ToxN sequences from various *E. coli* strains using MAFFT-DASH (19) (Supplementary Figure S1). While most sequences are well conserved (high sequence identity) in all strains, individual sequences show minor variations. Careful visual analysis showed that they might be grouped into five distinct sub-clusters (Clusters 1 to 5) and two single-membered clusters based on sequence variations within each cluster (Supplementary Figure S1). For ease of representation, we chose two representative sequences from each cluster and aligned them (Figure 1A). This alignment showed a set of conserved residues across all the clusters, which are likely to be important for the structure and function of the ToxIN system (Figure 1A) (discussed later).

As with the toxins, the antitoxin repeats seem to be unique in each cluster both in terms of the length and sequence of repeats. For example, the representative ToxI sequences from each cluster show that antitoxin consists of 4.6 repeats in cluster 1, 5.6 repeats in cluster 2, 4 repeats in cluster 3, 3 repeats in cluster 4, and about 3.8 repeats in cluster 5 (Figure 1B). We aligned the ToxI repeats of five representative sequences from the five main clusters identified (Figure 1C). The ToxI repeat sequence can be viewed in two parts: the core pseudoknot forming sequence and the flanking 5′ and 3′ single-stranded sequences. The alignment showed conservation of the pseudoknot forming region for the sequence and structure. However, the flanking 5′ and 3′ single-stranded regions varied across the sequences (Figure 1C). The structural and functional studies on type III TA systems from *P. atrosepticum*, *E. coli* and *B. thuringiensis* have revealed that ToxN protein cleaves A rich sequences, typically between two A nucleotides (6,7,11). The consensus cleavage sequences were found to be AA↓AU, GAA↓AU and AAA↓AA (where ↓ represents the position of cleavage) for the ToxIN systems from *P. atrosepticum*, *E. coli*, and the *B. thuringiensis*, respectively (6,7,11). The sequence alignment of ToxI repeats from the five *E. coli* clusters also suggested that the cleavage sequences are A rich; however, the toxin cleavage specificity may be unique for each cluster (with cluster 1 and 2 being similar) (Figure 1C).

### Type III toxin induces growth arrest in *E. coli*

Type III TA systems from other organisms, for example, *B. thuringiensis* and *P. atrosepticum*, could be functionally reconstituted in *E. coli* (5,7). Here, we have chosen to characterize a type III TA system from *E. coli* ToxIN cluster 1 (strain 680, assembly GCA_001893605.1) in laboratory strains of *E. coli* such as DH5α. This *E. coli* ToxN protein shares ∼80% sequence identity with previously characterized ToxN_Pa_ from *P. atrosepticum* (6). The sequence of the ToxN from *E. coli* GCA_001012275 that was used in a recent functional study differs by only one residue (Y to F at position 4) and belongs to cluster 1 (11). For simplicity, we refer to the system we have characterized in this study as just ToxIN or ToxIN_Ec_ if needed to distinguish from other ToxIN systems.

We used bacterial growth assays to assess the bactericidal or bacteriostatic nature of this system. For the growth assays, the type III toxin from *E. coli* strain 680 in the pBAD vector was transformed in *E. coli* DH5α cells. The expression of the protein was induced by the addition of 0.2% l-arabinose. The effect of overexpression of type III toxin on the growth of *E. coli* DH5α cells was quantified by measuring the optical density of the culture at 600 nm wavelength at regular intervals. As shown in Figure 1D, we observed that ectopic expression of type III toxin results in growth arrest in liquid cultures compared to the cells harboring vector-only control (see Materials and Methods). The growth assays were also performed on a solid media by spotting 10-fold serial dilutions of culture on LB agar plates (Figure 1E). It is evident again that the co-expression of protein toxin and RNA antitoxin restores the growth inhibition caused by the toxin. We also observed that the expression of ToxN protein led to a significant decrease (∼10^5^-fold) in the colony-forming units (CFU) of *E. coli* DH5α cells compared to the ToxN + ToxI control (Supplementary Figure S2), as previously reported for other type III TA systems (5,7,8). Overall, these results proved that the identified system functions as a typical type III TA system in *E. coli*.

### ToxIN_Ec_ forms a heterohexameric complex

**Figure 2.**
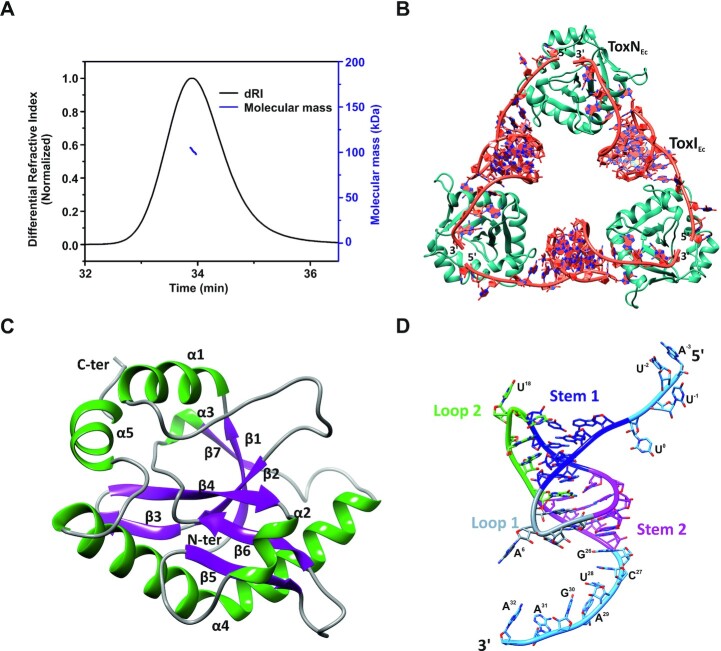
Crystal structure of the type III toxin–antitoxin complex from *E. coli*. (**A**) Oligomeric state and molecular mass analysis of the purified type III TA complex from *E. coli* using SEC-MALS. (**B**) 2.097 Å resolution crystal structure of the *E. coli* type III TA complex. The toxin protein (ToxN) is shown in teal and the antitoxin RNA (ToxI) is shown in dark orange. The crystal structure shows the arrangement of ToxIN complex in a cyclic heterohexameric assembly of alternating ToxN and ToxI. (**C**) Structure of ToxN protein as observed in the crystal structure of the TA complex. α-helices are shown in dark green and β-strands are depicted in purple. (**D**) Structure of ToxI RNA pseudoknot as observed in the ToxIN complex crystal structure. Stem 1 and Stem 2 of the pseudoknot are depicted in blue and magenta respectively. Loop 1 and Loop 2 are shown in grey and light green respectively and the single-stranded termini are shown in sky blue color. The nucleotides in the 5′ and 3′ single-stranded overhangs are marked.

The *E. coli* ToxIN complex was expressed and purified as described (see Materials and Methods). Briefly, the toxin and antitoxin were cloned in two different vectors, and the resultant plasmids were co-transformed for the co-expression of toxin and antitoxin. The first step of purification involved Ni-NTA—His-tag affinity chromatography followed by anion-exchange chromatography. Interestingly, we received three distinct elution peaks at different concentrations of NaCl in the ion-exchange chromatography step. These peaks corresponded to free protein (eluted at low salt concentration), RNA–protein TA complex (at intermediate salt concentration), and free antitoxin RNA (at high salt concentration). Therefore, we achieved purification of all three species of interest (free protein ToxN, ToxIN TA complex, and free antitoxin ToxI repeat) simultaneously. The presence of RNA and protein in the complex was confirmed using urea–PAGE and SDS-PAGE analysis, respectively, and the mass of the protein was verified by the LC–ESI-MS mass spectrometry (Supplementary Figure S3A–C). To ascertain the oligomeric state of the complex, the SEC-MALS experiment was performed, which provided a mass of ∼101.5 kDa corresponding to a heterohexamer consisting of three proteins and three RNA molecules (Figure 2A).

The purified complex was subsequently concentrated and crystallized. Crystals were obtained in several conditions; however, crystals obtained under a specific condition (see Materials and Methods) in *P*1 2_1_1 space group diffracted to the highest resolution of 2.097 Å at a synchrotron X-ray source (Table 1). The structure of the ToxIN_Ec_ complex was solved using the molecular replacement method using *P. atrosepticum* ToxIN_Pa_ complex (PDB ID 2XDB) structure as the search model (6). The asymmetric unit contained two hexameric assemblies of TA complexes consisting of six ToxN_Ec_ and six ToxI_Ec_ repeats (Supplementary Figure S4A, B). However, the biological assembly comprises of one heterohexameric unit consisting of three ToxN_Ec_ and three ToxI_Ec_ repeats (Figure 2B). The heterohexamer is a cyclic assembly of three proteins bound to three RNA arranged alternately in a head to tail manner. This is the highest resolution crystal structure of a type III TA complex that has been solved so far (Table 1).

The endoribonuclease toxin ToxN_Ec_ is a well-structured protein consisting of five α-helices and seven β-strands (Figure 2C). β-strands form the core of the protein that is supported by α-helices on the outside. There are two β-hairpin motifs formed by strands β 3–4 and β 5–6 and a kinked helix α4 that extends from Pro109 to Gln141. There is a cis-peptide formed by Gly37 that helps in the interaction of strands β1 and β2 (Figure 2C). Overall, the toxin has a fold very similar to some type II family RNase toxins, as has been observed previously (6,7).

**Figure 3.**
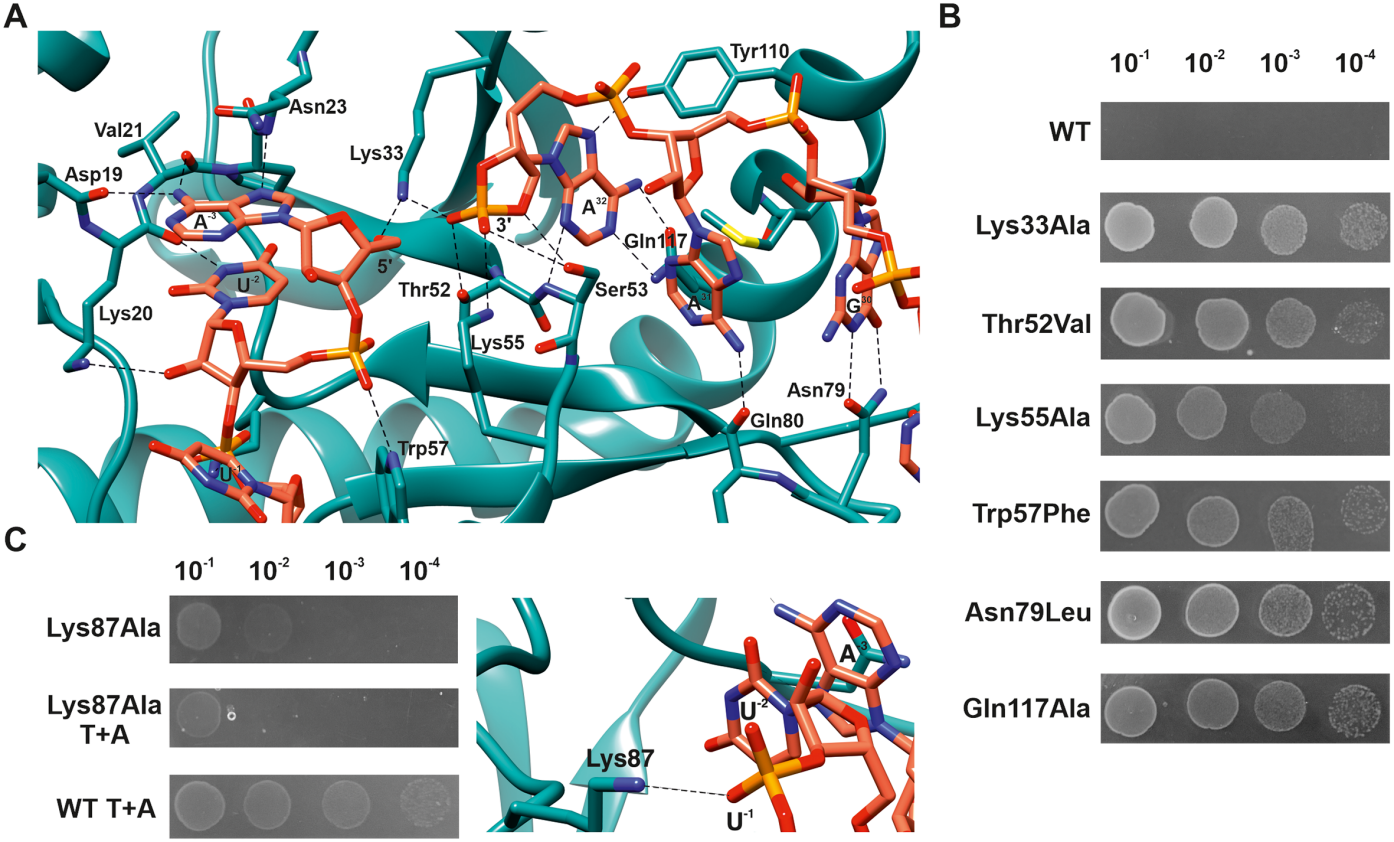
Residues important for ToxN activity and ToxN-ToxI interaction. (**A**) Interactions between ToxI and ToxN at the active site of ToxN. Atoms and bonds are represented as sticks with nitrogen, oxygen, and phosphorous atoms in blue, red and orange respectively. Hydrogen bonds are shown by black dashed lines. (**B**) ToxN activity probed *in vivo* by single point mutations of key residues. Spot growth assay of *E. coli* DH5α cells (by 10-fold serial dilutions) overexpressing WT ToxN and mutants. (**C**) Mutation of Lys87 residue of ToxN that interacts with ToxI backbone away from the active site destabilizes ToxN-ToxI interaction, as revealed by spot growth assays with Lys87Ala toxin and toxin + antitoxin (T+A).

The core of the antitoxin ToxI_Ec_ RNA repeat contains a pseudoknot, which is flanked by single-stranded regions on either side (5′ and 3′ single-stranded regions). The nucleotides in the ToxI_Ec_ are numbered as per the convention adopted previously (6). Therefore, the 5′ overhang, core pseudoknot, and 3′ overhang regions are present from −3 to 0, 1 to 25, and 26 to 32 nucleotide positions respectively in the functional ToxI_Ec_ repeat (Figure 2D). ToxI_Ec_ forms an H-type pseudoknot consisting of two stems (S1 & S2) and two loops (L1 & L2). The pseudoknot fold is stabilized by coaxial stacking of S1 and S2 and an intricate network of tertiary interactions (Figure 2D). Stem S1 consists of four canonical base pairs that are further stabilized by A-minor interactions by A19 and A20 from loop L2. The tertiary interactions of nucleotide G5 enable the loop L1 to turn sharply, connecting S1 and S2 and simultaneously positioning A6 to be able to interact with ToxN. S2 is formed by four base pairs, including a noncanonical U-U base pair between U12 and U22, which interacts with G21 from loop L2 to form a U:U:G triplet. The single-stranded overhangs constitute the recognition and cleavage sequence for ToxN protein. There is a series of sequence-specific interactions between ToxN and ToxI RNA terminal overhang regions (Figure 3A).

Interestingly, while analyzing the electron density of ToxI in the crystal structure, we found that the backbone atoms of the loop L2 region containing nucleotides U18-A20 to be flexible in an otherwise rigid RNA structure. The electron density for the U18 base was also not well defined in most of the ToxI chains, and the backbone electron density suggested the presence of two conformations (Supplementary Figure S4C). We incorporated the two conformations by splitting the RNA chain into two from nucleotides U18 to G21, which was validated by analyzing the B-factors. The corresponding loop L2 in the ToxI_Pa_ structure determined previously seemed to be stabilized with the help of metal ion binding to this region (6). The overall structures of ToxN_Ec_ and ToxI_Ec_ are similar to the toxins and the antitoxins from ToxIN_Pa_ and ToxIN_Bt_ complexes (6,7) (Supplementary Figure S5A-D).

In the TA complex, ToxI and ToxN interact extensively with stacking and H-bond interactions. There are several base-specific interactions by ToxI at the active site of ToxN involving nucleotides G30, A31, A32, A-3 and U-2 (Figure 3A). This structure provides evidence for sequence-specific (GAAAU) cleavage of ToxN_Ec_ at the molecular level. The residues Lys33, Thr52, Ser53, and Lys55 are found at the catalytically active site of ToxN, and the residues Trp57, Phe88, Tyr110 and Gln117 hold the RNA in place for ToxN to cleave the precursor ToxI. The electron density at the active site suggested that the 3′ terminal phosphate of ToxI cyclizes with the 2′-OH of A32 to form a cyclic phosphate, which is an intermediate in RNA cleavage reactions and has also been observed in the previously solved structures of type III TA complexes (6–8). The cyclic phosphate is stabilized by interactions with residues from ToxN at the active site. Moreover, there are additional sequence-specific interactions by both ToxI (nucleotides A1, A6, U7, U8, C27, U28) and ToxN (amino acid residues Ser65, Glu73, Asn79, Lys87, Asp108, Tyr115, Lys116, Arg122) with each other, away from the active site, that could provide specificity to the antitoxin for the toxin and impart stability to the complex.

We also probed the significance of some of the key ToxN residues responsible for the toxin activity and toxin–antitoxin interactions. We generated single point mutations of residues Lys33, Thr52, Lys55, Trp57, Asn79, Lys87 and Gln117 and assayed for toxin activity using *E. coli* spot growth assays. Expression of ToxN mutants ToxN Lys33Ala, Thr52Val, Lys55Ala, Trp57Phe, Asn79Leu and Gln117Ala resulted in the growth of *E. coli*, suggesting that these mutations led to the loss of ToxN toxicity (Figure 3B). However, mutation of residue Lys87 to Ala resulted in only a minor reduction in ToxN activity, which was not mitigated by the co-expression of ToxI (Figure 3C). This showed that some residues such as Lys87 are crucial for ToxN-ToxI interaction rather than ToxN endoribonuclease activity. As seen from the structure, Lys87 indeed interacts with the ToxI RNA; however, it is away from the active site of ToxN (Figure 3C).

### Conserved residue positions in *E. coli* ToxN and ToxI repeats

**Figure 4.**
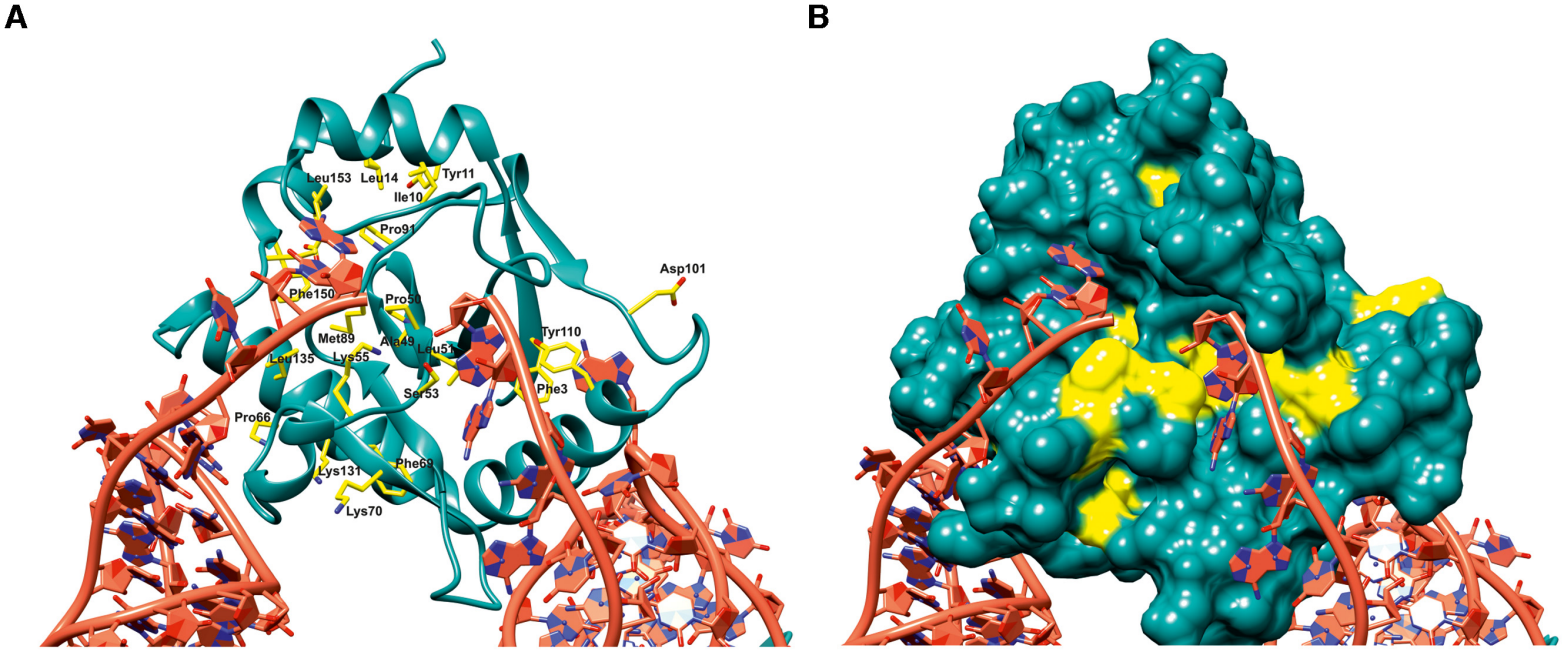
Structural and functional roles of conserved residues in ToxN. Multiple sequence alignment of ToxN proteins from all five ToxIN clusters in *E. coli* revealed several residues that are conserved in all clusters. (**A**) The conserved residues are marked and denoted in yellow in the ToxIN_Ec_ structure where ToxN_Ec_ is shown in cartoon representation and the conserved residues are shown as sticks. (**B**) Surface representation of ToxN_Ec_ where the conserved residues are coloured in yellow.

The sequence alignment of ToxN sequences revealed the presence of several residues that were absolutely conserved across all the five clusters (Figure 1C). The structure of ToxIN_Ec_ explains the potential significance of these residues (Figure 4A). Most of the conserved residues are found to be involved in maintaining the ToxN fold, ToxI interaction or ToxN catalytic activity (Figure 4A and B). The residues Phe3, Tyr10, Ile11, Leu14, Val39 are involved in hydrophobic interactions that stabilize the ToxN structure (Figure 4A). The conserved Gly37 forms a cis-peptide that helps in the interaction of strands β1 and β2, crucial for the ToxN fold. The conserved residues Ala49, Pro50, Leu51 near the active site that form the twisted strand β4 are likely to be responsible for holding the active site residues Thr52, Ser53 in the proper orientation for ToxN catalytic activity (Figure 4A). The catalytic residue Lys55 is also conserved across clusters, whereas Lys33 is substituted by similarly charged arginine in clusters 4 and 5 (Figure 1A).

The ToxI alignment showed that the core pseudoknot-forming region is well conserved among the clusters, whereas the 5′ and 3′ single-stranded regions are variable (Figure 1C). The RNA regions that form the two stems of the pseudoknot are structurally conserved, and the bases that are involved in tertiary interactions of the RNA structure are conserved even at the sequence level (Figure 1C). The two G–C base pairs in the middle of stem S1 are conserved across the clusters along with bases A19 and A20, which interact with these G–C base pairs through A-minor interactions. The nucleotides U12 and U22 that form the noncanonical U–U base pair as well as G21 that is part of the U:U:G triplet, are conserved in all the clusters. Base G5, which is crucial for ToxI folding, and base A6 that interacts with ToxN are conserved as well (Figure 1C).

### The core sequence of ToxI is sufficient for pseudoknot formation

**Figure 5.**
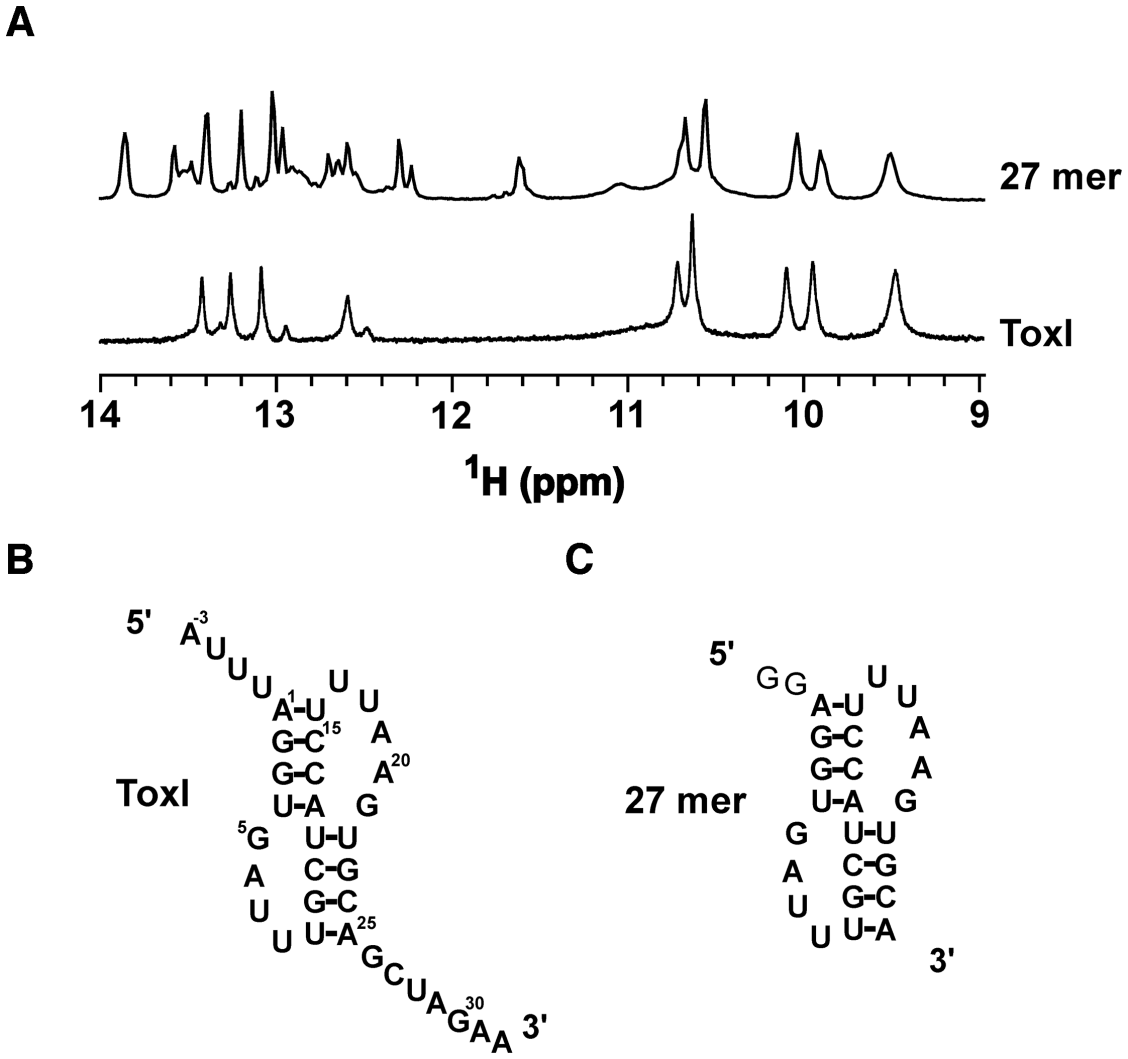
Structure of free ToxI probed by NMR. (**A**) Imino region of 1D ^1^H NMR spectra of ToxI and only pseudoknot forming region of a truncated 27mer ToxI. (**B, C**) The predicted secondary structures of free ToxI (**B**) and 27 mer RNA (**C**).

We wanted to understand if the core antitoxin RNA sequence can fold into a pseudoknot structure independent of toxin's binding. We purified the ToxI RNA repeat (from *E. coli*, along with the ToxN, and ToxIN complex) and recorded a 1D ^1^H NMR spectrum of the RNA (Figure 5A, B). From the ToxI pseudoknot structure in the ToxIN_Ec_ complex (Figure 2D), we predict nine hydrogen-bonded iminos resulting in at least nine peaks in the imino region of 1D ^1^H spectra. We observed about 12 peaks in the imino region of the 1D ^1^H spectra (Figure 5A). While this showed that the ToxI_Ec_ repeat must form a folded RNA structure, it also suggested the presence of more than one conformation for the ToxI RNA structure.

The 5′ and 3′ overhangs in the ToxI_Ec_ repeat form the major protein binding sides in ToxIN_Ec_ complex. Therefore, these overhang regions may influence the formation of the core pseudoknot. To understand the role or influence of 5′ and 3′ overhang regions on core pseudoknot formation and to verify that the imino peaks arise only from the core pseudoknot, we made a construct where we deleted the 5′ and 3′ overhangs to keep only the pseudoknot forming sequence in the RNA (27mer) (Figure 5C). We recorded a 1D ^1^H spectrum of the truncated RNA and overlaid it onto the spectrum of the complete ToxI repeat (Figure 5A). As shown in Figure 5A, the spectra of the two constructs overlaid very well, suggesting that the core structure is the same in both cases (Figure 5A).

### ToxN_Ec_ and ToxI_Ec_ interact with nanomolar affinity

The structures of the ToxIN complexes reported here or earlier have revealed a unique, closed cyclic RNA-protein complex structures (6,7). The ToxI RNA has two unique ToxN binding sites, i.e. at the 5′ and 3′ end of ToxI RNA repeats. While type III TA complexes have been purified and crystallized before, the binding affinities of these complexes in terms of dissociation constant and binding energetics has not been reported.

**Figure 6.**
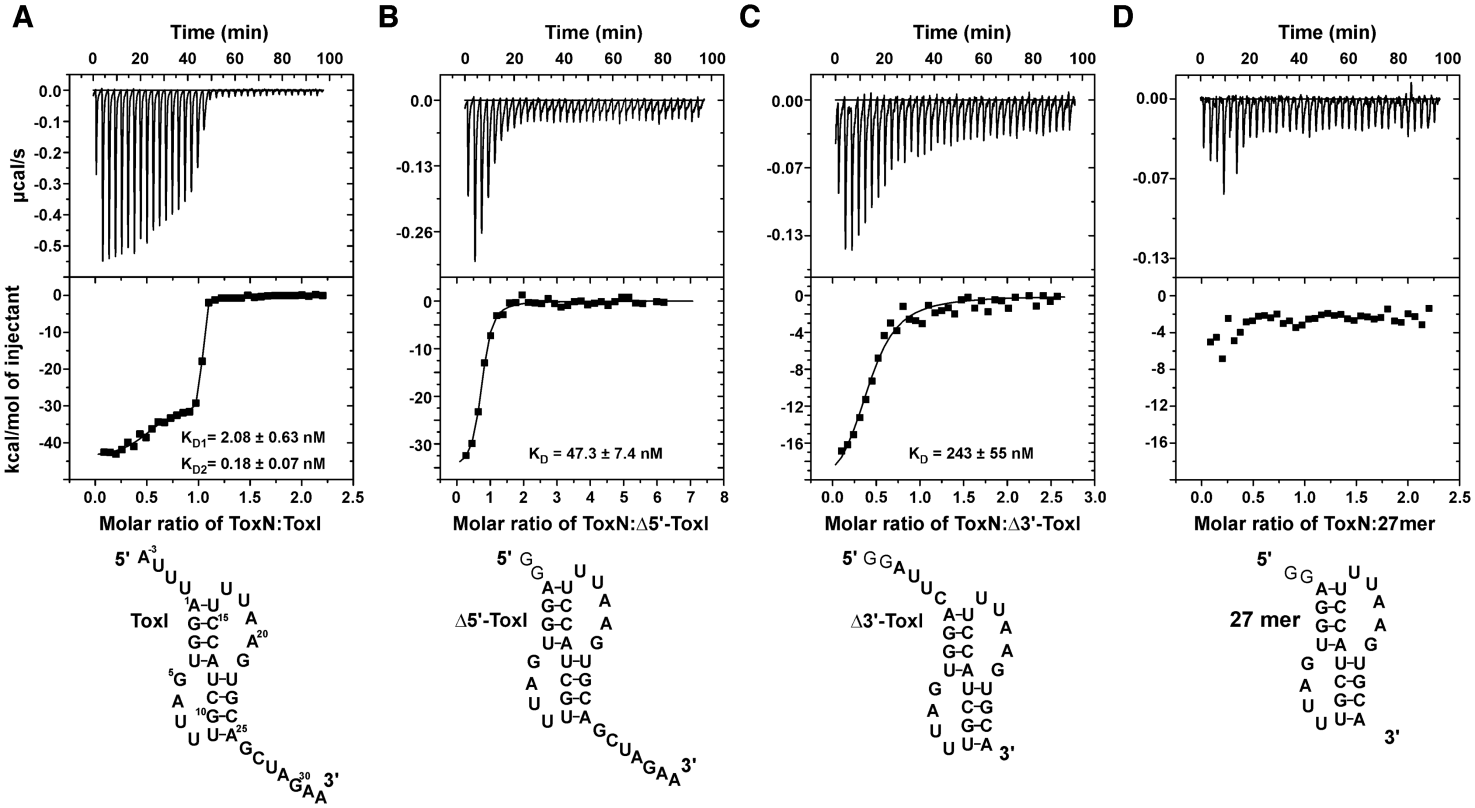
Interaction of ToxN protein with ToxI RNA monitored by isothermal titration calorimetry (ITC). (A–D) ITC isotherms of titration of ToxN protein with ToxI (**A**), Δ5′-ToxI (**B**), Δ3′-ToxI (**C**) and 27mer (**D**) RNAs. Raw and fitted ITC isotherms are shown and the *K*_D_ values obtained upon fitting the data are mentioned in the figures. The predicted secondary structures of the free RNAs are shown below the corresponding ITC profiles.

We used the isothermal titration calorimetry (ITC) method to characterize the binding of toxin and antitoxin to form the ToxIN_Ec_ complex. ITC experiments provide a complete thermodynamic profile that includes determination of equilibrium dissociation constant (*K*_D_), enthalpy change (Δ*H*), entropy change (Δ*S*) and stoichiometry (*n*) of interaction under a given experimental condition. The purified ToxN_Ec_ protein in the cell was titrated with purified ToxI_Ec_ RNA repeat, and the experiments were performed 2–3 times for data consistency. On titrating toxin with antitoxin, we repeatedly observed a biphasic curve corresponding to two sites and two-step enthalpically driven binding (Figure 6A). The first binding event corresponds to binding affinity with a *K*_D_ value of 2.08 nM, which was followed by a second higher affinity binding with a *K*_D_ value of 0.18 nM (Table 2). The two-step binding of ToxN_Ec_ to ToxI_Ec_ is commensurate with the crystal structure of the ToxIN_Ec_ complex that showed that the protein toxin binds to the 5′ and 3′ ends of antitoxin RNA uniquely to generate the cyclic hetero-hexameric complex. The two single-stranded termini of the antitoxin RNA that interact with the toxin are different both in terms of the sequences and the length of the single-stranded region. This two-step binding of the toxin to antitoxin is due to the fact that the two binding sites are non-equivalent.

**Table 2.** Equilibrium dissociation constants and other thermodynamic parameters derived for ToxN–ToxI interactions

Experiment		*K*_D_ (nM)	Δ*G* (kcal/mol)	Δ*H* (kcal/mol)	*T*Δ*S* (kcal/mol)
**ToxN–ToxI**	**1^st^ step**	2.08 ± 0.63	−11.47 ± 3.46	−30.25 ± 0.98	−18.78 ± 5.66
	**2^nd^ step**	0.18 ± 0.07	−12.99 ± 4.94	−44.38 ± 1.00	−31.39 ± 11.95
**ToxN–Δ5′-ToxI**		47.3 ± 7.4	−9.69 ± 1.52	−35.75 ± 0.98	−26.06 ± 4.10
**ToxN–Δ3′-ToxI**		243 ± 55	8.74 ± 1.98	−22.51 ± 1.83	−13.77 ± 3.12

To further confirm this, we designed two truncated ToxI antitoxin repeats. The 5′ and 3′ single-stranded overhangs in ToxI repeat were removed to generate Δ5′-ToxI (nucleotides −3 to 0 removed) and Δ3′-ToxI (26–32 removed) RNAs using *in vitro* transcription (Figure 6B and C). The ITC experiments performed using two truncated RNAs revealed that ToxN protein interacts with both the RNAs with high affinity. ToxN binds to Δ5′-ToxI RNA with a *K*_D_ of ∼47.3 nM and with Δ3′-ToxI RNA with a *K*_D_ of ∼243 nM (Figure 6B and C and Table 2). Therefore, these results prove that the 3′ single-stranded overhang containing ToxI RNA binds to ToxN with approximately five times better binding affinity than the 5′ single-stranded overhang containing ToxI (Table 2). We also performed an ITC experiment of the ToxN with only core pseudoknot containing RNA (27 mer) to determine if the pseudoknot alone can interact with ToxN. However, no appreciable heat change was observed, suggesting that only core pseudoknot is not sufficient for stable TA complex formation and the single-stranded regions of ToxI are essential for ToxN binding (Figure 6D). Further, we titrated a DNA oligonucleotide of the same sequence as the ToxI repeat (ToxI-DNA) with the ToxN protein. However, no significant heat change for the binding was observed (Supplementary Figure S6), showing that ToxN does not bind to the single-stranded DNA.

## DISCUSSION

*E. coli* is the most genetically tractable model organism available to decipher the mechanism of bacterial cellular processes. A plethora of genetic and molecular biology tools are available for *E. coli* that makes it a default model organism to carry out a detailed functional genetic analysis (30). Therefore, identification of type III TA systems in *E. coli* is important and would lead to functional analysis of these systems in greater depth. Recently, Guegler and Laub have reported the identification, and functional characterization of the first *E. coli* type III TA system (11). Using high-throughput RNA-seq analysis, authors have shown that the ToxN protein blocks the T4 phage protein synthesis by cleaving viral transcripts. Phage-induced shutoffs of *E. coli* transcription were shown to be necessary and sufficient to liberate ToxN (11).

Based on the sequence analysis reported here, we have found that the ToxIN systems in different *E. coli* strains can be classified into at least five distinct clusters. The toxin ToxN across all the clusters shows remarkable conservation of key residues that are important for the structure and endoribonuclease activity of the protein. In ToxI RNA repeats, the central pseudoknot forming sequences is highly conserved in terms of the sequence and the structure with unique flanking 5′ and 3′ overhangs sequences suggesting distinct binding and cleavage specificity of ToxN for RNA substrate in each cluster.

The type II toxin–antitoxin systems are overall a well-studied class of TA systems. The toxin and antitoxin in type II TA systems are protein molecules (1). The inhibition of toxin by direct binding by antitoxin is common in type II and type III TA systems. Several type II toxins are sequence-specific endoribonucleases (31,32). Similarly, the ToxN toxin is also an endoribonuclease. However, it is a special endoribonuclease that also processes its own cognate antitoxin RNA into single repeats besides specifically cleaving cellular or viral RNA upon activation. Protein ToxN binds and assembles with the ToxI repeats to form a catalytically inactive TA complex. The *E. coli* ToxIN complex structure reported here and previously studied ToxIN complexes from *P. atrosepticum, E. rectale* and *B*. *thuringiensis* have revealed a detailed view of the catalytic center of type III toxins (6–8). Recent studies have shown that *E. coli* type III toxin ToxN has a cleavage preference for GAAAU sequence, where the toxin cleaves the sequence between third and fourth A nucleotides (6,11). The *E. coli* ToxIN complex structure reported in this study, revealed that ToxN protein recognizes the 5′ and 3′ overhangs of ToxI in a base-specific manner and also provided the structural basis for this sequence specificity. Since ToxN is a sequence-specific endoribonuclease, we also verified its activity through *in vitro* RNase assays (Supplementary Figure S7). The substrate used in this assay (dimer-ToxI) was derived from ToxI and consisted of two pseudoknot forming sequences that are connected via the ToxN cleavage sequence. Incubation of this RNA with ToxN showed a concentration-dependent specific cleavage of RNA (Supplementary Figure S7).

From the structure, it is clear that the 3′ overhang of ToxI binds to ToxN with a greater number of interactions than the 5′ overhang. This was validated by the ITC results that clearly showed that the 5′ overhang deleted ToxI repeat binds to ToxN with approximately 5-fold better affinity than the 3′ overhang deleted ToxI repeat. Only core pseudoknot (without the single-stranded 5′ and 3′ overhangs) was not able to form a stable complex with ToxN (Figure 6D). However, the structure of the ToxIN complex revealed that apart from the sequence-specific binding of the 5′ and 3′ overhangs, the base of the pseudoknot at both ends also interacts with the ToxN creating a high-affinity ToxIN complex. The kinked helix α4 is crucial for this interaction of ToxN with the edges of pseudoknot on opposite sites of two adjacent repeats. Therefore, the pseudoknot containing ToxI makes it a unique substrate for ToxN. ToxN can cleave and process the precursor ToxI RNA into single repeats, after which it binds the individual ToxI repeats and forms a stable and catalytically inactive ToxIN complex. In the case of its cellular RNA targets, however, it will likely bind, cleave, and release the cleaved products. Higher-order secondary structures near the cleavage site in cellular or viral RNA can therefore influence the rate of RNA cleavage.

The antitoxin protein in type II TA systems contains a labile, intrinsically disordered protein domain. Upon activation of the TA system under stress, several proteases such as lon proteases have been shown to be upregulated, which specifically cleave the labile antitoxin protein, thereby releasing the free toxin (33). In type III TA systems, the antitoxin RNA is structured with a pseudoknot in the ToxIN complex. Using NMR spectroscopy, we have shown that the antitoxin ToxI repeat forms pseudoknot structure in solution without ToxN protein. The central sequence forms the pseudoknot without the 5′ and the 3′ overhang regions. The stability of the pseudoknot structure has been shown to be important for ToxIN complex assembly. For example, mutation of intercalated G23, i.e. part of U:U:G triplet, showed reduced ability of ToxI_Bt_ to rescue bacterial growth in kill/rescue assays (7). These results show the importance of pseudoknot structure in regulating the activity of ToxIN systems. Identification of host or viral factors that can bind and destabilize ToxI pseudoknot remains elusive.

**Figure 7.**
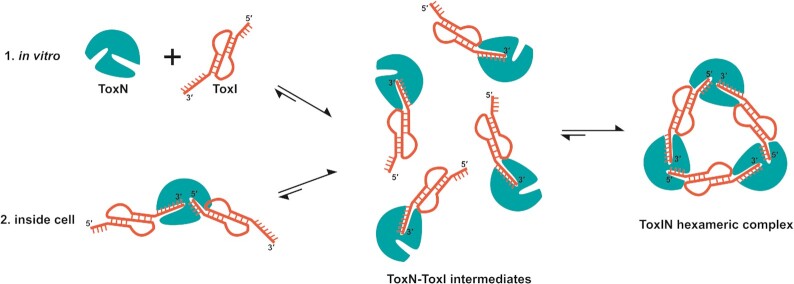
Plausible model of assembly of closed, heterohexameric ToxIN complex. The model suggests that the final closed, heterohexameric ToxIN complex is assembled from individual ToxN and ToxI units via a dimeric ToxN–ToxI intermediate.

Another interesting question specific to type III TA systems is how the toxin and antitoxin assemble to form a cyclic complex. ToxIN complex was proposed to be a dynamic complex in the cell (34). Hexameric ToxIN complex was suggested to be in equilibrium with the linear, heterogeneous ToxIN complexes in the cell, though there has been no evidence for the presence of any multimeric species of ToxI and ToxN other than the heterohexamer. However, the ITC results presented in our study suggest a strong affinity of ToxI RNA and ToxN protein in the ToxIN complex. Given that ToxN must process the full-length antitoxin RNA into individual repeats before the TA assembly, based on our ITC results, we have proposed a plausible model for the assembly of the ToxIN complex (Figure 7). In the ITC experiments, when ToxN is added to single ToxI repeats, it would initially associate with the 3′ end of ToxI, which has a higher binding affinity for ToxN to form a heterodimeric intermediate complex. In this intermediate state, the 5′ end of ToxI would be free. Subsequently, the three units of the intermediate complex assemble using the 5′ end of ToxI to form a cyclic heterohexameric ToxIN complex. This reflects as two-step binding in the ITC thermogram (Figure 6A). Similarly, inside the cell, once ToxN cleaves the full-length ToxI into individual repeats, it would release the 5′ end of processed ToxI repeats in some linear complexes while remaining bound to the 3′ end to form the dimeric intermediates, which would subsequently form the cyclic hexameric ToxIN complex (Figure 7). Both the pathways (in vitro and inside cell assembled) result in the final ToxIN complex of similar size as revealed by the size-exclusion chromatography (Supplementary Figure S8). Direct activation of ToxIN complexes in bacteria by disassembly of the high-affinity ToxN and ToxI complexes is unknown. Further studies, to ascertain the mechanism of ToxIN complex assembly and disassembly unambiguously, are necessary.

Under normal homeostatic growth conditions, the toxin remains bound to antitoxin as an inactive TA complex that gets activated under stress conditions such as phage infection (35). To prevent any accidental activation of the TA system, the antitoxin in type II TA systems usually acts as an auto-repressor, which is a characteristic feature of type II TA systems (36). Almost all the antitoxins in type II TA systems contain a DNA binding domain via which the antitoxin specifically binds to DNA sequences in the promoter/operator region of the TA operon (1,37). Whether type III TA systems are further regulated directly by ToxI or ToxN or other host or viral factors remains to be seen. Previous studies have proposed that factors from bacteriophage may prevent ToxIN activation or keep the ToxN protein inactive even after its release from the ToxIN complex (11,38–40).

Specific activation of TA systems in pathogenic bacteria is envisioned as a potential, novel antibacterial strategy (9). A clear understanding of TA complex assembly and structure can lead to the development of novel small molecules or peptide-based inhibitors of TA assembly, with the potential to develop them further as possible antibacterial drugs.

## DATA AVAILABILITY

The atomic coordinates and structure factors for the *E. coli* type III ToxIN complex have been deposited in the Protein Data Bank under PDB accession code 7D8O.

## ABBREVIATIONS

TA, toxin–antitoxin; NMR, nuclear magnetic resonance; ITC, isothermal titration calorimetry

## Supplementary Material

gkab1264_Supplemental_Files

## Notes

Present address: Sankaran Sandhya, Department of Biotechnology, Faculty of Life and Allied Health Sciences, MS Ramaiah University of Applied Sciences, Bengaluru 560054, India.
