# Supplementary material for: Identification, functional characterization, assembly and structure of ToxIN type III toxin–antitoxin complex from E. coli
Source: Nucleic Acids Res. 2022 Jan 8;50(3):1687–700. doi: 10.1093/nar/gkab1264 (PMC8860590; doi:10.1093/nar/gkab1264)
Supplement: gkab1264_Supplemental_Files [file gkab1264_supplemental_files.zip › Manikandan_etal_NAR_SI_rev_241221.pdf]

Supporting Information for:

Identification, functional characterization, assembly and structure of ToxIN type III toxin-antitoxin complex from *E. coli*

Parthasarathy Manikandan[1], Sankaran Sandhya[1], Kavyashree Nadig[1], Souradip Paul[1], Narayanaswamy Srinivasan[1], Ulli Rothweiler[2,*], and Mahavir Singh[1,*]

[1]Molecular Biophysics Unit, Indian Institute of Science, Bengaluru, 560012, India

[2]The Norwegian Structural Biology Centre, Department of Chemistry, The Arctic University of Norway, N-9037 Tromsø, Norway

Present address: Sankaran Sandhya, Department of Biotechnology, Faculty of Life and Allied Health Sciences, MS Ramaiah University of Applied Sciences, Bengaluru 560054, India.

This file contains:

1. Supplementary Figures S1 to S8
2. Supplementary Table S1 *(Table S2 is uploaded separately as an excel file)*.

Figure S1. Multiple sequence alignment of type III ToxIN toxins from different strains of *E. coli*. *E. coli* strains harbouring ToxN sequences belonging to five clusters defined in this study are depicted in distinct colours (cluster 1 in blue; cluster 2 in red, cluster 3 in magenta, cluster 4 in yellow, and cluster 5 in green colours). The ToxIN system that is studied in this study belongs to cluster 1. Two sequences (WP_032650368.1 and WP_002488419.1) are lone sequences representing single-membered clusters.

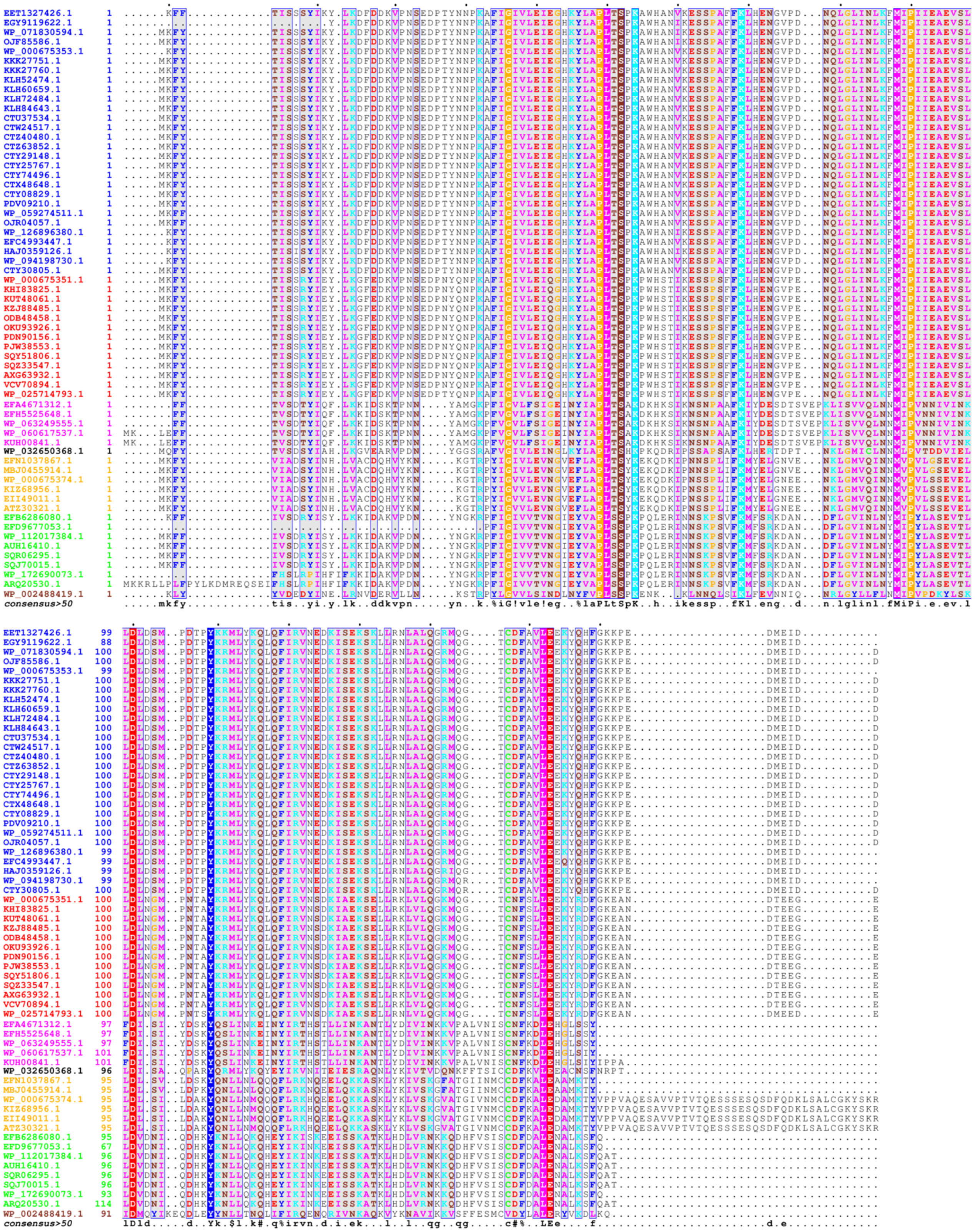

Figure S2. Colony forming units (CFU) of *E. coli* DH5α cells upon overexpression of *E. coli* type III toxin. CFU reduces significantly (~10^5-fold) upon expression of $ToxN_{Ec}$ as compared to co-expression of $ToxN_{Ec}$ and $ToxI_{Ec}$

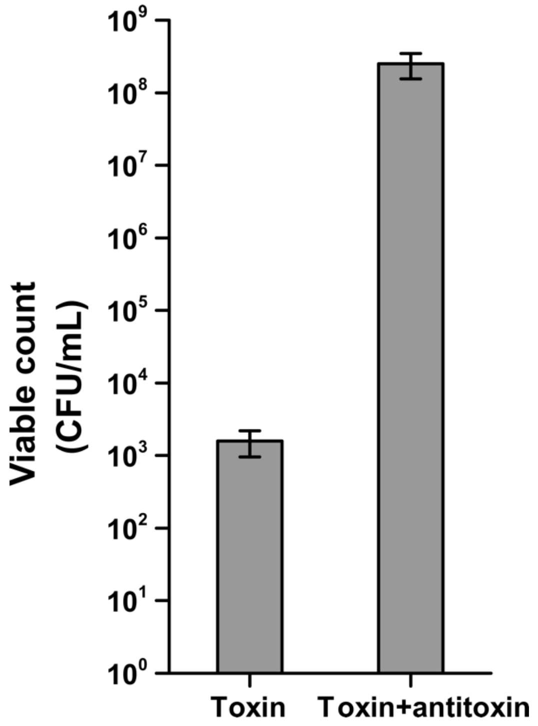

Figure S3. Characterization of ToxN and ToxI in purified ToxIN complex. A) SDS-PAGE analysis ToxIN complex. Gel is stained using Coomassie stain for protein to show the presence of ToxN protein. B) Urea-PAGE analysis ToxIN complex. Gel is stained using Toluidine blue dye for RNA to show the presence of ToxI RNA repeat. C) ESI-MS analysis of ToxN confirms the presence of protein of correct molecular weight.

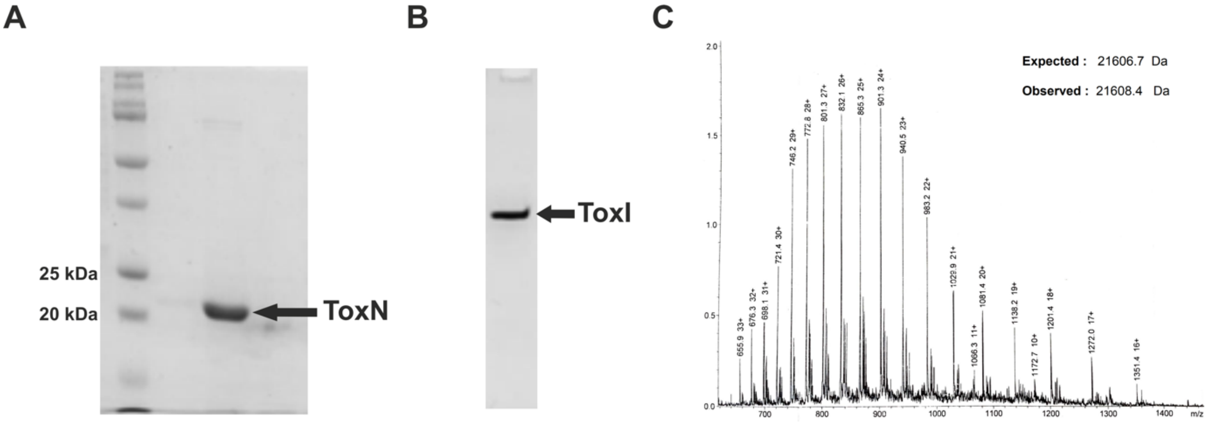

Figure S4. The content of the asymmetric unit of ToxIN crystals. A, B) The asymmetric unit comprises of two heterohexameric assemblies. Two views of the structures are shown (A and B). The biological assembly consists of one heterohexameric ToxIN complex as shown by SEC-MALS analysis. C) Side-view of the part of ToxIN complex where one ToxI molecule bound to two ToxN molecules are shown. The inset shows the electron density of nucleotides U18 to G21. The electron density of ToxI backbone revealed that the RNA backbone has two conformations between nucleotides U18 and A20.

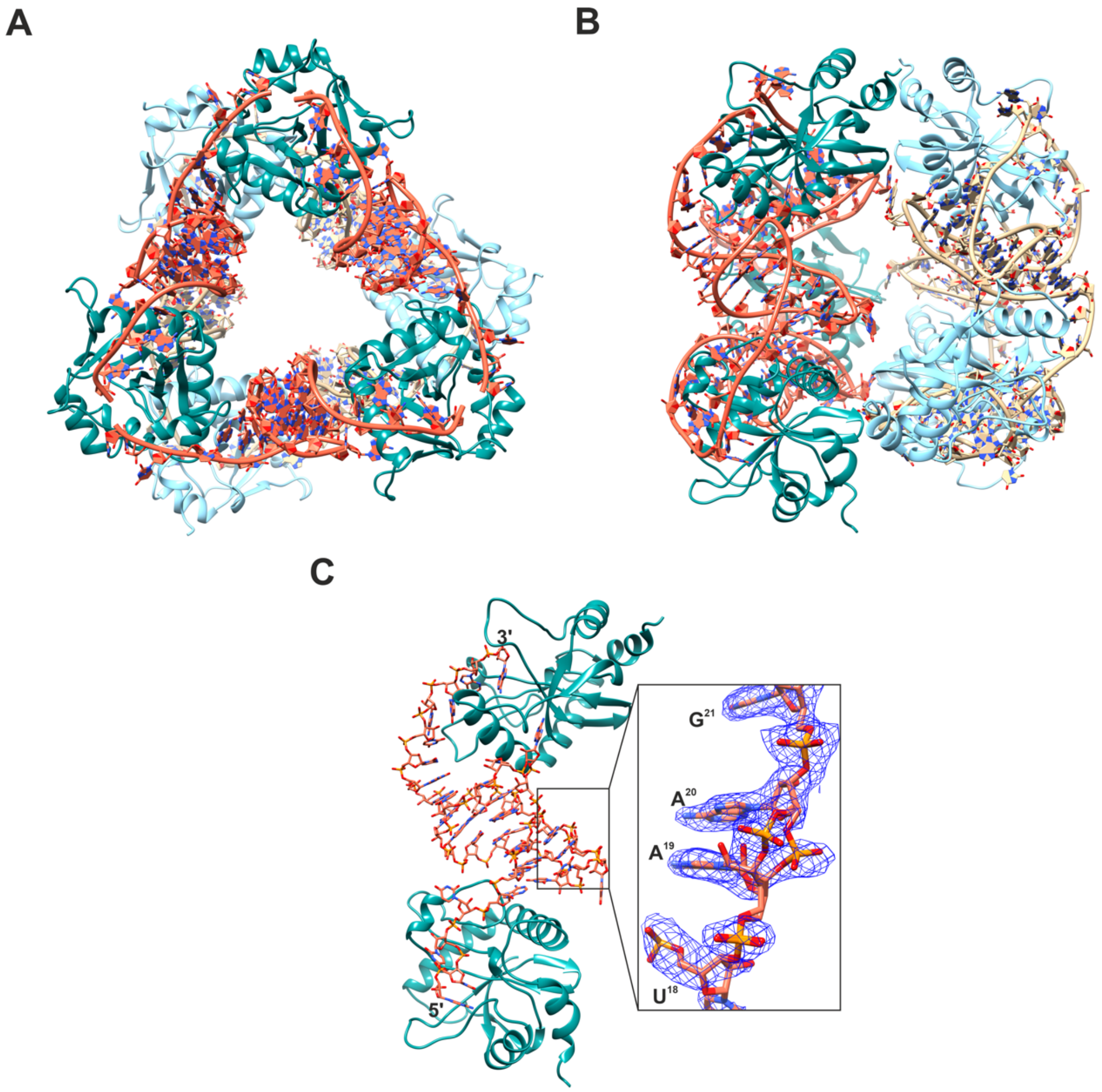

Figure S5. Comparison of *E. coli* ToxIN with existing structures of ToxIN systems. A) Overlay of ToxN structures from *E. coli* and *P. atrosepticum* (PDB ID 2XDB) ToxIN complexes. B) Overlay of ToxI structures from *E. coli* and *P. atrosepticum* (PDB ID 2XDB) ToxIN complexes. C) Overlay of ToxN structures from *E. coli* and *B. thuringiensis* (PDB ID 4ATO) ToxIN complexes. D) Overlay of ToxI structures from *E. coli* and *B. thuringiensis* (PDB ID 4ATO) ToxIN complexes.

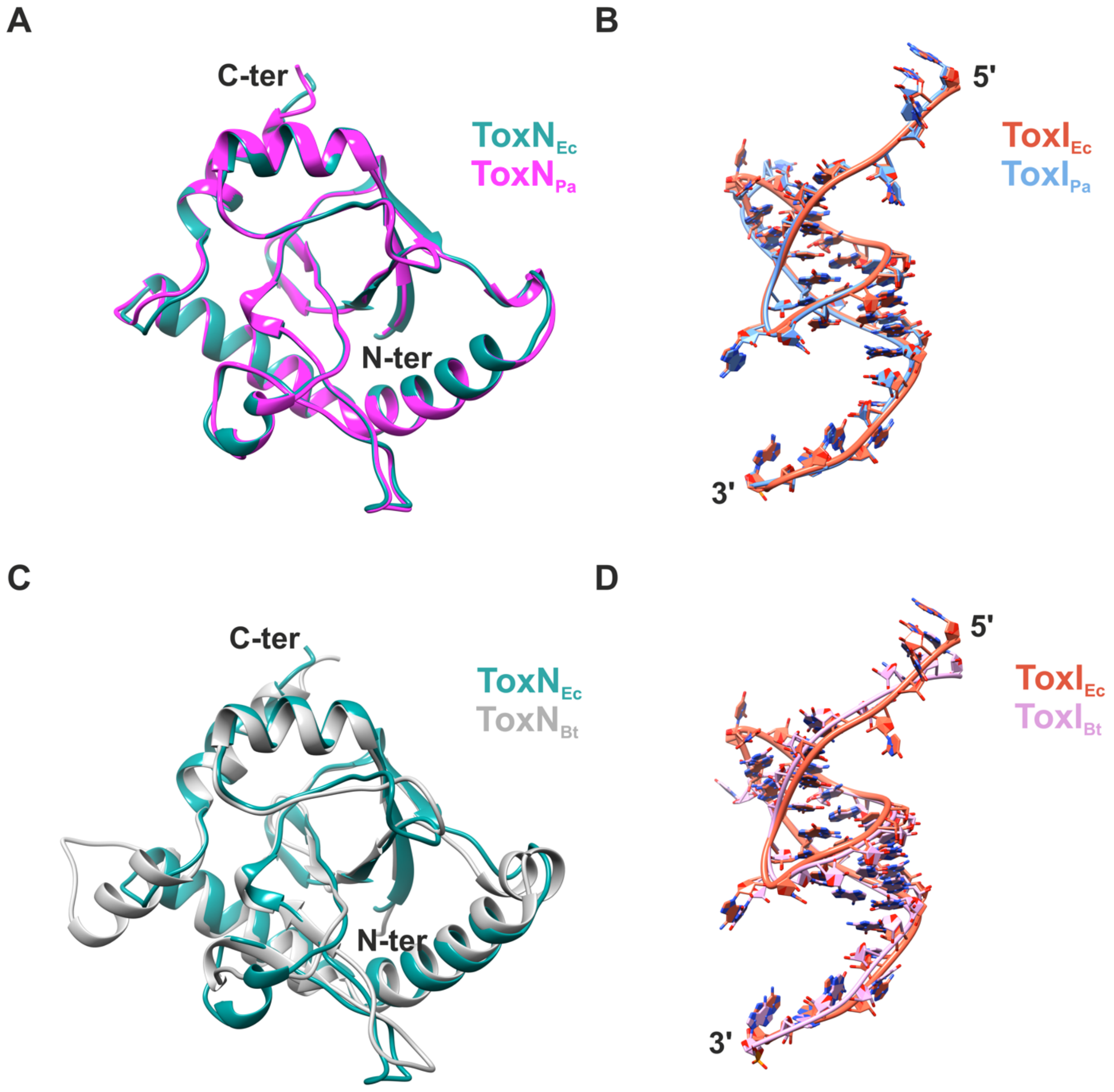

Figure S6. ToxN does not bind to DNA of same sequence as ToxI RNA. ITC experiments of ToxN with DNA oligonucleotide (ToxI-DNA) of the same sequence as antitoxin ToxI RNA did not show appreciable heat change suggesting that ToxN does not bind to DNA.

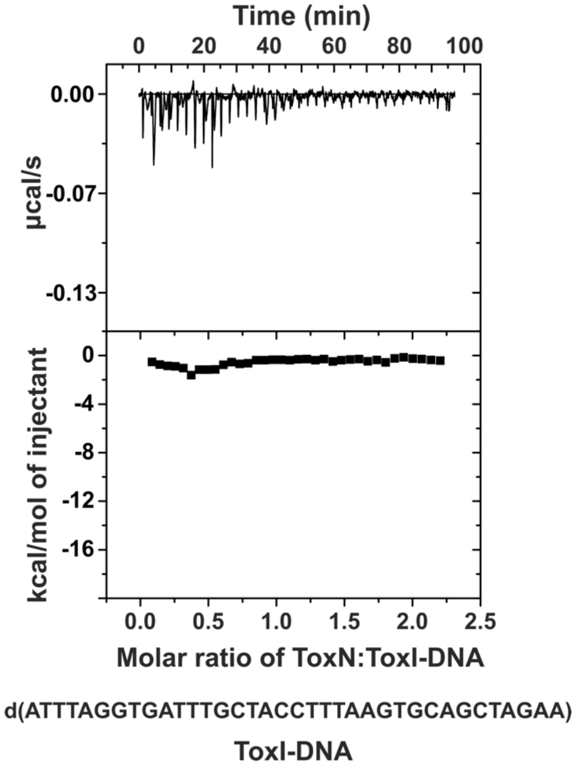

Figure S7. Purified ToxN$_{Ec}$ cleaves RNA *in vitro*. (A, B) Purified ToxN protein was incubated with an RNA substrate (dimer-ToxI) containing the consensus cleavage site in the single stranded region at 37°C. Analysis by urea-PAGE showed that ToxN cleaved dimer-ToxI RNA distinctly. (C) Predicted secondary structure of the substrate dimer-ToxI.

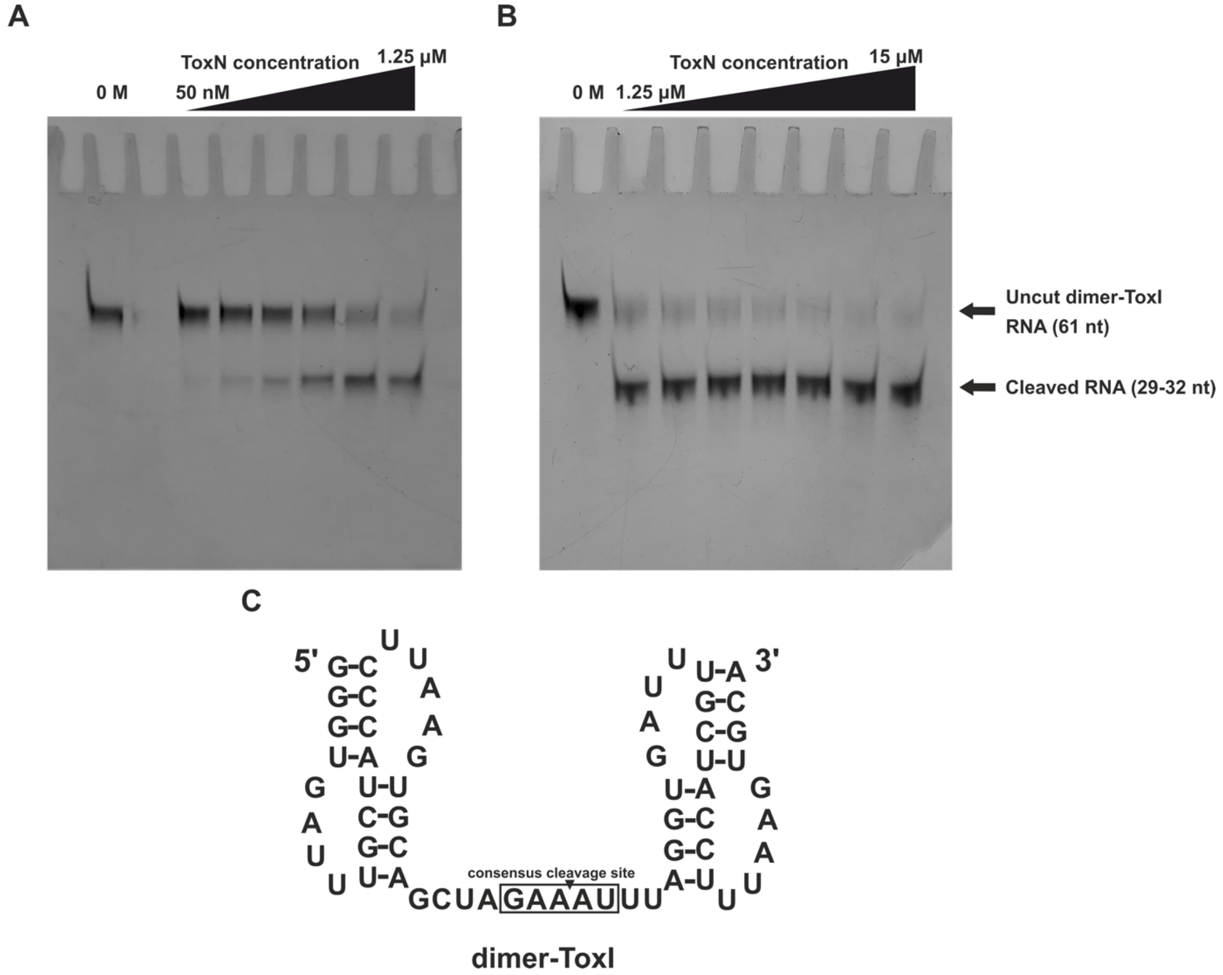

Figure S8. ToxN and ToxI assemble to form heterohexameric complex both *in vitro* and *in vivo*. Size Exclusion Chromatography (SEC) profiles of *in vivo* purified ToxIN$_{Ec}$ complex and ToxIN$_{Ec}$ complex formed in vitro (sample taken after the ITC titration) overlay well suggesting that same heterohexameric assembly is formed in both cases.

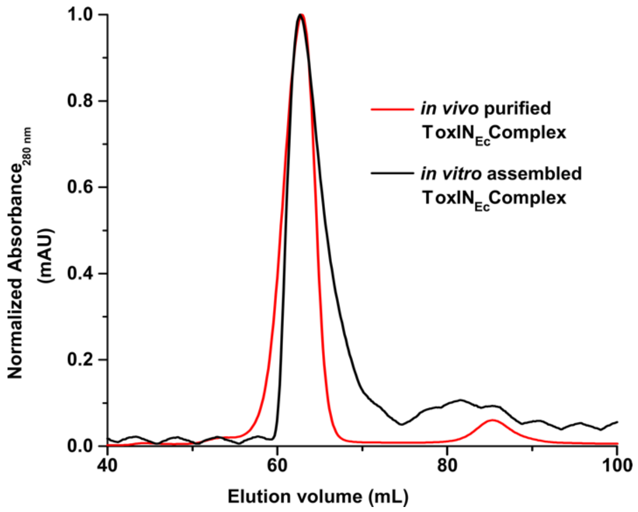

Table S1. Sequences of primers and DNA templates used for in vitro transcription of RNA sequences.

**Cloning and mutagenesis primers**	
Ectoxin_Fwd	AGGTCATATGGCGAAGTTCTTTACCATCAGC
Ectoxin_Rev	ATGCTCTAGACTCGAGGTCGTCAATCTCC
pRSF_Fwd	CGGACACTCGAGCTTAATTAACCTAGGCTGC
pRSF_Rev	GCAATCCCATGGCTAATGCAGGAGTCGC
EctaiiiRNA_Fwd	CTAAATCCATGGCACCTAGTTGTAAGCCCAAGC
EctaiiiRNA _Rev	CGACTACTCGAGGGTATATCTCCTTCTTTTTTCTCGT
Lys33Ala_Fwd	AACCCGGCGGCGTTCATCGG
Lys33Ala_Rev	GAACGCCGCCGGGTTGTTGTAGG
Thr52Val_Fwd	CGCTGGTCAGCCCGAAGGC
Thr52Val_Rev	GGGCTGACCAGCGGCGCC
Lys55Ala_Fwd	AGCCCGGCGGCGTGGCACGCG
Lys55Ala_Rev	CCACGCCGCCGGGCTGGTCAGC
Trp57Phe_Fwd	AAGGCGTTCCACGCGAACGTGAAAGAGAGC
Trp57Phe_Rev	GCGTGGAACGCCTTCGGGCTGGTCAGC
Asn79Leu_Fwd	CCGGACCTCCAGCTGGGCCTGATCAACC
Asn79Leu_Rev	AGCTGGAGGTCCGGAACACCGTTTTCG
Lys87Ala_Fwd	AACCTGGCATTTATGATTCCGATCATTGAGG
Lys87Ala_Rev	CATAAATGCCAGGTTGATCAGGCCCAGC
Gln117Ala_Fwd	TATAAAGCGCTGCAATTCATCCGTGTTAACG
Gln117Ala_Rev	TTGCAGCGCTTTATACAGCATACGC
**Sequences of DNA template for *in vitro* transcription of RNA**	
27mer	TGCACTTAAAGGTAGCAAATCACCTCCTATAGTGAGTCGTATTAG
Δ5’-ToxI	TTCTAGCTGCACTTAAAGGTAGCAAATCACCTCCTATAGTGAGTCGTATTAG
Δ3’-ToxI	TGCACTTAAAGGTAGCAAATCACCTGAATCCTATAGTGAGTCGTATTAG
dimer-ToxI	TGCACTTAAAGGTAGCAAATCACCTAAATTTCTAGCTGCACTTAAGGGTAGC AAATCACCCTATAGTGAGTCGTATTAG
Prom19 sequence	CTAATACGACTCACTATAG
